# Transcript profile analysis reveals important roles of jasmonic acid signalling pathway in the response of sweet potato to salt stress

**DOI:** 10.1038/srep40819

**Published:** 2017-01-13

**Authors:** Huan Zhang, Qian Zhang, Hong Zhai, Yan Li, Xiangfeng Wang, Qingchang Liu, Shaozhen He

**Affiliations:** 1 Beijing Key Laboratory of Crop Genetic Improvement/Laboratory of Crop Heterosis and Utilization, Ministry of Education, China Agricultural University, Beijing 100193, China

## Abstract

Sweet potato is an important food and bio-energy crop, and investigating the mechanisms underlying salt tolerance will provide information for salt-tolerant breeding of this crop. Here, the root transcriptomes of the salt-sensitive variety Lizixiang and the salt-tolerant line ND98 were compared to identify the genes and pathways involved in salt stress responses. In total, 8,744 and 10,413 differentially expressed genes (DEGs) in Lizixiang and ND98, respectively, were involved in salt responses. A lower DNA methylation level was detected in ND98 than in Lizixiang. In both genotypes, the DEGs, which function in phytohormone synthesis and signalling and ion homeostasis, may underlie the different degrees of salt tolerance. Significant up-regulations of the genes involved in the jasmonic acid (JA) biosynthesis and signalling pathways and ion transport, more accumulation of JA, a higher degree of stomatal closure and a lower level of Na^+^ were found in ND98 compared to Lizixiang. This is the first report on transcriptome responses to salt tolerance in sweet potato. These results reveal that the JA signalling pathway plays important roles in the response of sweet potato to salt stress. This study provides insights into the mechanisms and genes involved in the salt tolerance of sweet potato.

Salinity stress is becoming a major constraint on crop production; it affects approximately 19.5% of the irrigated soil worldwide, and will result in a loss of up to 50% of cultivable land by the middle of the twenty-first century^1^,^2^. High concentrations of salts lead to ion imbalances and hyperosmotic effects and cause a number of physiological disorders in plants, including photosynthesis inhibition, metabolic dysfunctions and cellular structure damage^3^. Therefore, developing salt-tolerant cultivars has become one of the most important goals in crop breeding.

Phytohormones play important roles in mediating host responses to various abiotic stresses. Increasing evidence has shown that jasmonic acid (JA, or the related compound methyl jasmonate, MeJA) functions in diverse physiological processes in plants, such as the regulation of plant responses to abiotic stresses^4^,^5^,^6^,^7^,^8^,^9^,^10^,^11^. The exogenous application of JA to wheat seedlings for three days has been shown to significantly enhance the salt tolerance of these plants by increasing the activities of antioxidant enzymes and the concentrations of antioxidative compounds^4^. MeJA improves salinity resistance in German chamomile by increasing the activity of antioxidant enzymes^5^. The endogenous JA content has been shown to be markedly increased under drought and cold stresses but reduced under heat stress in rice^6^. Expression profiling and physiological characterization on salinity stress response in barley reveal the JA-mediated adaptation to salinity stress^7^. Zhao *et al*. showed that a wheat allene oxide cyclase (AOC) gene enhances salinity tolerance via the JA signalling pathway^8^. The steady-state levels of two key enzymes in JA biosynthesis, lipoxygenase (LOX) and allene oxide synthase (AOS), and the content of JA are higher in salt-tolerant tomato cultivars^9^. The salt hypersensitivity of the *lox3* mutant can be complemented by MeJA^10^. *VaNAC26* enhances drought tolerance by improving JA biosynthesis in Arabidopsis^11^. Stomatal closure induced by abscisic acid (ABA) and JA is a fundamental physiological response in plants^12^. Calcium-dependent protein kinase 6 (*CPK6*) functions as a positive regulator of MeJA signalling to activate ion channels in Arabidopsis guard cells^13^. Oxylipins serve as signalling molecules to control stomatal closure in a JA-dependent manner to enhance the defence response in Arabidopsis^14^. ABA is another mediator of plant responses to a variety of stresses, and this hormone plays a critical role in regulating the water status and growth of plants via the induction of genes encoding enzymes and other proteins involved in cellular dehydration tolerance^15^. Moreover, ABA is involved in adaptations to salt and drought in rice, tobacco and other plants^16^,^17^,^18^. Salicylic acid (SA) plays an indeterminate role in defence mechanisms against abiotic stresses^19^. The application of exogenous SA has been shown to enhance the drought and salt tolerance of bean and tomato^20^, whereas pre-treatment with SA has been shown to decrease drought tolerance in maize^21^.

Illumina paired-end sequencing technology is an important method for accessing genomic data sources for gene discovery and functional studies. High-throughput sequencing of transcriptomes has been performed for many model and non-model plant species, such as Arabidopsis, rice, maize, wheat and cotton^3^,^22^,^23^,^24^,^25^. Sweet potato (*Ipomoea batatas* (L.) Lam.) is an important root and tuber crop and is used as an industrial and bio-energy resource. This crop has considerable potential to grow on saline land^26^,^27^, and several salt tolerance genes have been identified in sweet potato^28^,^29^,^30^,^31^,^32^. Recently, *de novo* whole-genome sequencing of the selfed line Mx23Hm and the highly heterozygous line 0431-1 of *I. trifida* (2n = 2x = 30, one of the likely diploid ancestors of sweet potato) was performed on an Illumina HiSeq platform, but the assembly and annotation of these genomes are still in the early stages^33^. Yan *et al*. reported the complete chloroplast genome sequence of sweet potato variety Xushu 18^34^. However, the sweet potato genome is still unavailable due to its highly heterozygous, generally self-incompatible, outcrossing and autohexaploidy nature. To date, the transcriptome sequencing of sweet potato has provided important transcriptional data for studying storage root formation and starch, carotenoid and anthocyanin biosynthesis and characterizing the associated key genes in this crop^27^,^35^,^36^,^37^; however, transcriptome sequencing studies of sweet potato under abiotic or biotic stresses are rare.

In the present study, RNA-Seq was used to identify salt tolerance-related genes and to determine their crosstalk in the roots of the salt-sensitive sweet potato variety Lizixiang and the salt-tolerant line ND98 under salt stress. Transcript profile analysis indicated that the JA signalling pathway is involved in the responses of sweet potato to salt stress. Decreased DNA methylation may also contribute to the salt tolerance of this crop. The present study provides a better understanding of how sweet potato responds to salt stress and has potential applications for the genetic improvement of sweet potato on marginal land.

## Results

### Transcriptome sequencing of Lizixiang and ND98

**Figure 1:**
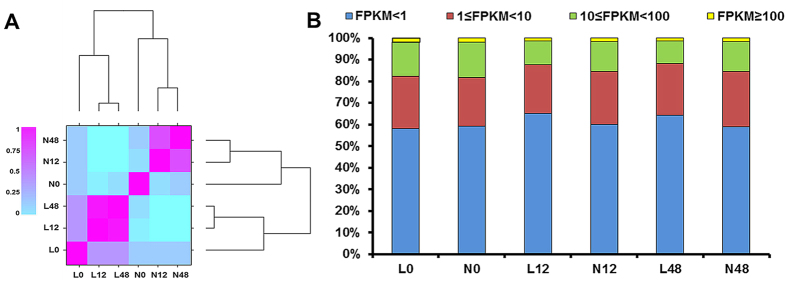
Gene expression analysis of the Lizixiang and ND98 root transcriptomes. (**A**) Correlation of the global gene expression in Lizixiang and ND98 roots under 0-, 12-, and 48-h salt treatments. (**B**) Statistics of the gene expression levels (FPKM) in Lizixiang and ND98 samples. L0, Lizixiang 0 h; L12, Lizixiang 12 h; L48, Lizixiang 48 h; N0, ND98 0 h; N12, ND98 12 h; and N48, ND98 48 h.

*In vitro*-grown Lizixiang and ND98 plants were assessed using RNA-Seq analysis in two independent biological replicates. RNA samples were collected from the roots at 0, 12, and 48 h after exposure to 200 mM NaCl stress for RNA-Seq. In total, 368,444,980 clean reads were obtained from the 12 RNA-Seq datasets, and 230,357 transcripts, with a mean length of 1,298 bp, were *de novo* assembled using Trinity (Fig. 1A, Supplementary Table S1). The transcripts were further clustered into 63,673 non-redundant transcripts (unigenes) with a mean length of 953.5 bp (Supplementary Table S1). For functional annotation, the sequences of the assembled unigenes were searched among a variety of databases, and 37,016 unigenes were annotated with putative functions based on hits from at least one database (Supplementary Table S2). In addition, when the sequences of the assembled unigenes were compared to the genome sequences of *I. trifida* (2n = B_1_B_1_ = 2x = 30)^33^, a low pair alignment rate of 51.70% was obtained. This might be because the assembly of the *I. trifida* (2x) genome is still in the early stages and sweet potato is a highly heterozygous autohexaploid (2n = B_1_B_1_B_2_B_2_B_2_B_2_ = 6x = 90).

### Identification and functional annotation of differentially expressed genes

Fragments per kilobase of transcript per million mapped reads (FPKM) was used to represent the gene expression levels generated using TopHat. The proportion of genes with FPKM ≥ 1 was 2.5% (671 genes) lower in ND98 than in Lizixiang under normal conditions (0 h), whereas the proportion was 13.9% (3099 genes) and 15.2% (3466 genes) higher in ND98 than in Lizixiang under 12 and 48 h of salt stress, respectively (Fig. 1B). The differentially expressed genes (DEGs) of Lizixiang and ND98 were detected via pair-wise comparisons at each of the two time points. In total, 8,744 and 10,413 DEGs were detected in Lizixiang and ND98, respectively (Table 1, Supplementary Table S3).

Using the K-means method, 8,744 and 10,413 DEGs of Lizixiang and ND98, respectively, were divided into 10 clusters based on their expression patterns (Supplementary Fig. S1). A total of 2,819 DEGs showed the same expression patterns between Lizixiang and ND98, and 7299 DEGs exhibited different expression patterns. Further analysis indicated that the expression levels of 1238 DEGs were significantly higher in ND98 than in Lizixiang for at least one time point during salt treatment; these genes were associated with signal transduction, ion transport, cell wall synthesis, disease resistance, photosynthesis processes, reactive oxygen species (ROS) scavenging and some unknown functions (Supplementary Table S4).

**Figure 2:**
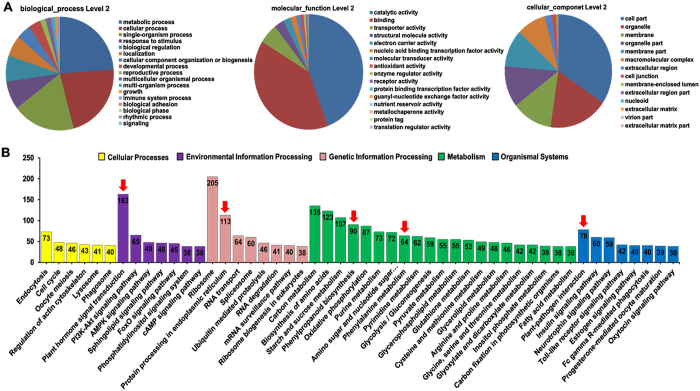
Functional enrichment of genes with different expression patterns between Lizixiang and ND98. (**A**) GO functional classification analysis of genes with different expression patterns between the two genotypes (p < 0.05). (**B**) Top 50 KEGG pathways enriched with genes with different expression patterns between the two genotypes. The pathways related to phytohormone biosynthesis and signalling are indicated with red arrows.

Using Gene Ontology (GO) analysis, the 7299 DEGs with different expression patterns between Lizixiang and ND98 were classified into three categories: biological process, molecular function and cellular component. In the biological process class, 20 biological processes with significant differences in enrichment were identified, including “metabolic processes” (3,542 DEGs), “cellular processes” (3,270 DEGs), “single-organism processes” (2,780 DEGs), “responses to stimuli” (1,207 DEGs) and other processes. In the molecular function class, “binding” (3,338 DEGs) and “electron carrier activity” (2,984 DEGs) were significantly enriched. In the cellular component class, “cell part” was the most significantly enriched GO term (2581 DEGs) (Fig. 2A).

To explore the biological pathways important for sweet potato responses to salt stress, the DEGs with different expression patterns between Lizixiang and ND98 were further annotated to the reference pathways in the Kyoto Encyclopedia of Genes and Genomes (KEGG) using KeggArray software (Fig. 2B). These DEGs were assigned to 258 KEGG pathways. The pathway search results were sorted based on the number of hits, and the ribosome pathway (205 members) and plant hormone signal transduction (163 members) were the most highly represented groups. The genes involved in protein processing in the endoplasmic reticulum (113 members), phenylpropanoid biosynthesis (90 members), plant-pathogen interactions (78 members) and phenylalanine metabolism (64 members) were also hallmarks of sweet potato roots under salt stress (Fig. 2B).

**Figure 3:**
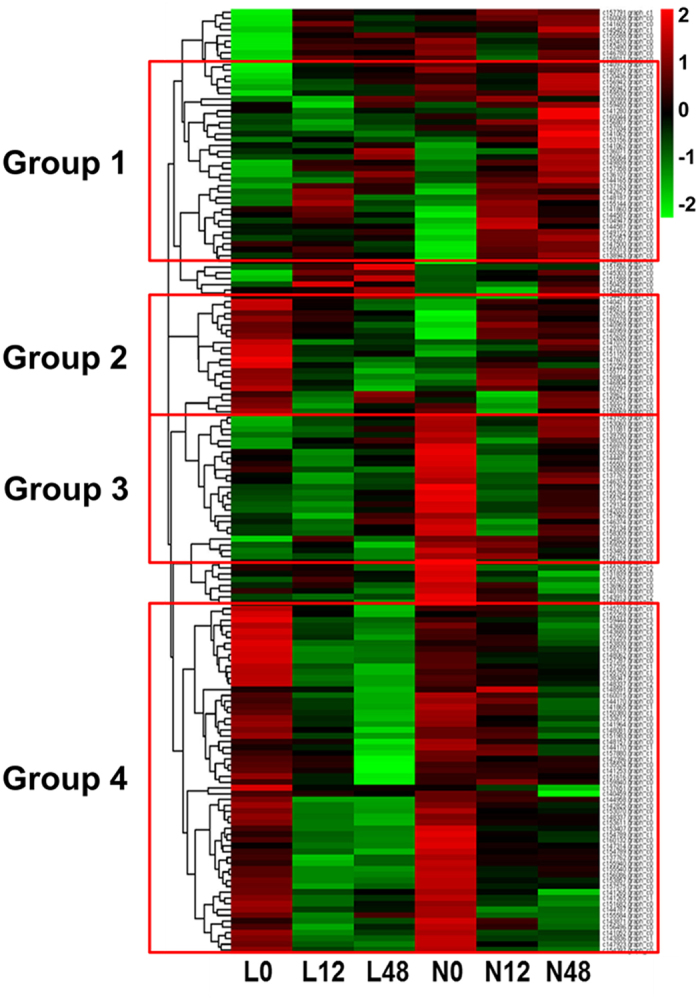
Heat maps of DEGs with different expression patterns between Lizixiang and ND98 that participate in phytohormone signalling pathways. Group 1 genes showed low basal expression in both Lizixiang and ND98 under normal conditions but were induced under salt stress only in ND98. Group 2 genes exhibited high and low basal expression in Lizixiang and ND98, respectively, and were suppressed and induced under salt stress in Lizixiang and ND98, respectively. Group 3 genes were highly expressed only in ND98 under normal conditions but were not induced under salt stress in either genotype. Group 4 genes were highly expressed in both genotypes under normal conditions but were induced under salt stress only in ND98. L0, Lizixiang 0 h; L12, Lizixiang 12 h; L48, Lizixiang 48 h; N0, ND98 0 h; N12, ND98 12 h; and N48, ND98 48 h.

Phytohormone synthesis- and transport-related genes in the roots of both genotypes were identified and categorized into four groups via gene expression profiling (Fig. 3). The first group showed low basal expression in both Lizixiang and ND98 under normal conditions, but these genes were induced under salt stress only in ND98. The second group exhibited high and low basal expression in Lizixiang and ND98, respectively, and these genes were suppressed and induced under salt stress in Lizixiang and ND98, respectively. The genes in the third group were highly expressed only in ND98 under normal conditions, but were not induced under salt stress in either genotype. The genes in the fourth group were highly expressed in both genotypes under normal conditions, but were induced under salt stress only in ND98 (Fig. 3). These results suggest that the two genotypes have different mechanisms underlying the phytohormone synthesis and transduction pathways in response to salt stress.

### Analysis of DNA methylation

**Figure 4:**
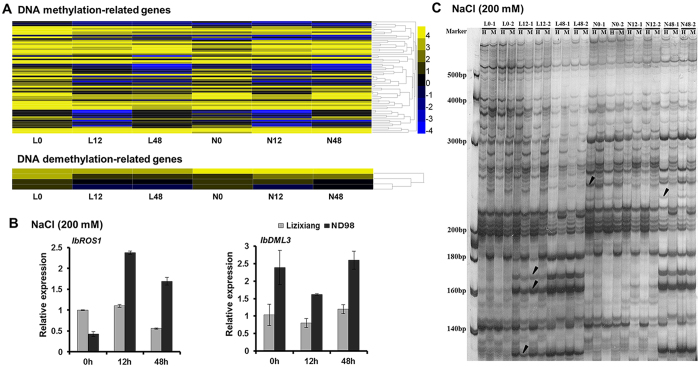
Differences in the global genomic DNA methylation in Lizixiang and ND98 under salt stress. (**A**) Heat maps showing the expression of the genes related to DNA methylation or demethylation. (**B**) Differential expression of DNA demethylation-related genes validated using qRT-PCR. *IbROS1*, c144343.graph_c0; *IbDML3*, c148603.graph_c0. (**C**) MSAP profiling analysis showing different patterns of locus-specific DNA methylation in Lizixiang and ND98. The gel shows the results for one primer combination; twenty *EcoRI* + *HpaII*/*MspI* primer combinations were used in total. H, *HpaII*; M, *MspI.* Differentially methylated sites between Lizixiang and ND98 are indicated with arrows. L0, Lizixiang 0 h; L12, Lizixiang 12 h; L48, Lizixiang; N0, ND98 0 h; N12, ND98 12 h; and N48, ND98 48 h.

DNA methylation influences global gene expression under abiotic stresses in plants^38^. All DNA methylation- and demethylation-related genes were identified via gene expression profiling under salt stress (Fig. 4A, Supplementary Table S5). The expression level of the key gene associated with methylation, DNA methyltransferase DNMT1-like enzyme (*IbMET1*), was higher in ND98 than in Lizixiang, but three other key gene types, domains rearranged methyltransferase 1 (*IbDRM1*), domains rearranged methyltransferase 2 (*IbDRM2*) and chromomethylase 2 (*IbCMT2*), did not show differences between the two genotypes (Supplementary Table S6). Both key demethylation-related genes, namely DNA demethylases demeter-like protein (*IbDML3*) and DNA repressor of silencing 1 (*IbROS1*), exhibited higher expression levels in ND98 than in Lizixiang (Fig. 4B, Supplementary Table S6).

A methylation-sensitive amplified polymorphism (MSAP) analysis was performed to detect methylation variations at specific sites in Lizixiang and ND98 root samples (Fig. 4C). Twenty *EcoRI* + *HpaII*/*MspI* primer combinations yielded 731, 744 and 718 clear and reproducible sites in Lizixiang root samples and 767, 858 and 717 clear and reproducible sites in ND98 root samples at 0, 12 and 48 h, respectively (Table 2). The proportions of unmethylated (pattern *HpaII*/*MspI* = 1/1), fully methylated (pattern *HpaII*/*MspI* = 0/1) and hemi-methylated (pattern *HpaII*/*MspI* = 1/0) CCGG sites in Lizixiang and ND98 root samples are listed in Table 2. The Lizixiang root samples presented 225, 295 and 266 methylated CCGG sites and the ND98 root samples presented 215, 255 and 251 methylated CCGG sites at 0, 12 and 48 h, respectively, revealing higher demethylation in the ND98 samples than in the Lizixiang samples (Table 2).

The enhanced expression of DNA methylation- and demethylation-related genes was accompanied by changes in the DNA methylation and demethylation levels. Under salt stress, lower DNA methylation levels were accompanied by a larger proportion of FPKM ≥ 1 genes in ND98 than in Lizixiang (Fig. 1B, Table 2). Both genotypes showed variations in epigenetic modifications, particularly DNA methylation, which in turn influenced the expression of stress tolerance-related genes in response to salt stress.

### Response of JA, ABA and SA signalling pathways to salt stress

**Figure 5:**
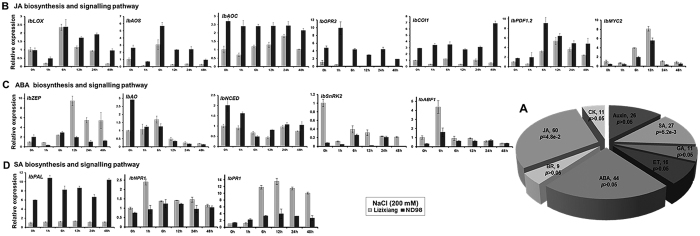
Distribution and expression of the genes in the JA, ABA and SA biosynthesis and signalling pathways. (**A**) Analysis of the distribution of genes in phytohormone signalling pathways. The *P* values were determined using singular enrichment analysis (http://bioinfo.cau.edu.cn/agriGO). The top three categories of phytohormones were JA, ABA and SA, accounting for 64.2% of the DEGs in the hormone class. ABA, abscisic acid; BR, brassinosteroid; CK, cytokinins; ET, ethylene; GA, gibberellin; JA, jasmonic acid; and SA, salicylic acid. (**B**), (**C**) and (**D**) Expression of the key genes in the JA, ABA and SA biosynthesis and signalling pathways, respectively, under salt stress. *IbLOX*, c154376.graph_c0; *IbAOS*, c110075.graph_c0; *IbAOC*, c136730.graph_c0; *IbOPR3*, c151632.graph_c0; *IbCOI1*, c148337.graph_c2; *IbPDF1.2*, c136835.graph_c0; *IbMYC2*, c143913.graph_c0; *IbZEP*, c158900.graph_c0; *IbAO*, c150446.graph_c0; *IbNCED*, c156411.graph_c0; *IbSnRK2*, c148195.graph_c0; *IbABF1*,c125138.graph_c1; *IbPAL*, c147773.graph_c3; *IbNPR1*, c151920.graph_c0; *IbPR1*, c160535.graph_c0.

Genes with different expression patterns between the two genotypes and implicated in different phytohormone signalling pathways were identified under salt stress. The top three categories, in terms of phytohormones, were JA, ABA and SA, accounting for 64.2% of the phytohormone signalling-related genes (Fig. 5A, Supplementary Table S7). The expression of sweet potato homologs of key genes involved in JA, ABA and SA signalling pathways was validated using real-time quantitative PCR (qRT-PCR)^8^,^9^,^39^,^40^,^41^,^42^. The JA biosynthesis and signalling genes lipoxygenase (*IbLOX*), allene oxide synthase (*IbAOS*), OPDA reductase 3 (*IbOPR3*), coronatine-insensitive 1 (*IbCOI1*) and plant defensin gene 1.2 (*IbPDF1.2*) were significantly induced under salt stress in ND98, and the expression levels of these genes were significantly higher in ND98 than in Lizixiang after exposure to salt stress (Fig. 5B). The expression levels of ABA biosynthesis genes zeaxanthin epoxidase (*IbZEP*), aldehyde oxidase (*IbAO*) and 9-cis-epoxycarotenoid dioxygenase (*IbNCED*) showed changes under salt stress in both genotypes, but these genes were not induced in ND98 under salt stress (Fig. 5C). The SA biosynthesis gene phenylalanine ammonia-lyase (*IbPAL*) was up-regulated, but other biosynthesis and signalling genes were not induced under salt stress in ND98 (Fig. 5D). The expression patterns of these genes were consistent with the results of the RNA-Seq analysis (Supplementary Table S6). These results indicated that the JA signalling pathway was involved in responses to salt stress in ND98.

**Figure 6:**
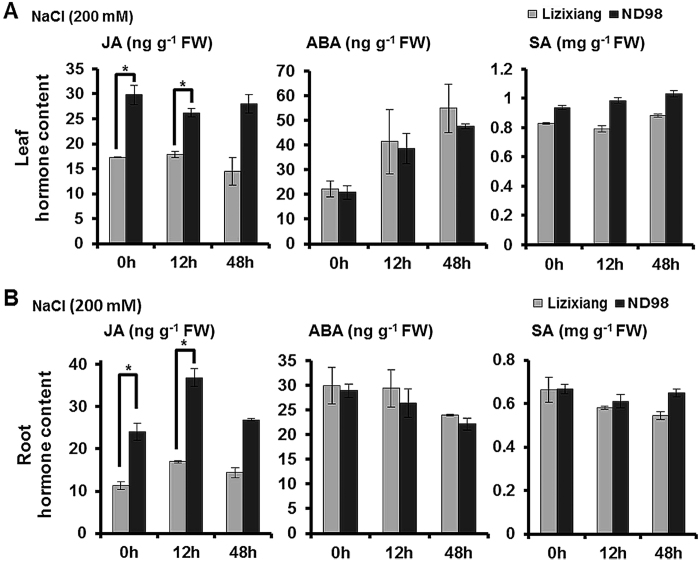
Responses of JA, ABA and SA in the roots and leaves of Lizixiang and ND98 under salt stress. (**A**) JA, ABA and SA contents in the leaves. (**B**) JA, ABA and SA contents in the roots. The data are presented as the means ± SD (n = 3). *P ≤ 0.05 for significant differences between Lizixiang and ND98 according to Student’s t-test.

To verify the expression patterns of the genes involved in phytohormone metabolism, the contents of endogenous JA, ABA and SA were measured in the roots and leaves of Lizixiang and ND98. Significant differences were not observed in either the ABA or the SA contents in the leaves or roots between Lizixiang and ND98 before or after salt stress treatment (Fig. 6A,B). Though the JA content was significantly higher in the leaves and roots of ND98 than in Lizixiang under normal conditions, the JA content in the ND98 roots was significantly increased after 12 h of salt stress compared to that of untreated ND98, while the JA content in the Lizixiang roots showed no significant difference before or after salt stress treatment, indicating that JA signalling pathway can be activated by salt stress in ND98 (Fig. 6A,B). These results further showed that JA plays a crucial role in the enhanced salinity tolerance of ND98 relative to that of Lizixiang plants.

### Analysis of stomatal conductance and transpiration rate

**Figure 7:**
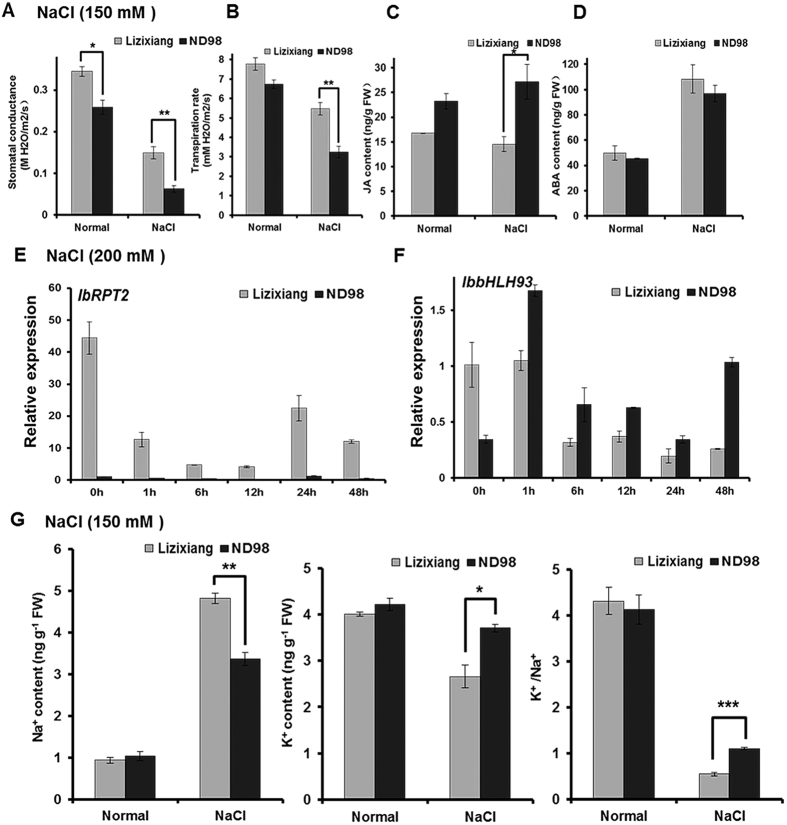
Different stomatal opening and closing phenotypes and Na^+^ and K^+^ contents in Lizixiang and ND98. (**A**) Stomatal conductance. (**B**) Transpiration rates. (**C**) JA content. (**D**) ABA content. (**E**) The expression levels of stomatal opening-related gene *IbRPT2* validated using qRT-PCR. *IbRPT2*, c158200.graph_c0. (**F**) The expression levels of stomatal closure-related gene *IbbHLH93* validated using qRT-PCR. *IbbHLH93*, c152736.graph_c0. (**G**) Na^+^ and K^+^ content and K^+^/Na^+^ ratio. The data are presented as the means ± SD (n = 3). *P ≤ 0.05, **P ≤ 0.01, and ***P ≤ 0.001 indicate significant differences between Lizixiang and ND98 (Student’s t-test).

Stomatal conductance and transpiration rate are important parameters for evaluating the degree of stomatal opening; these parameters are negatively correlated with stomatal closure^43^. Accordingly, the stomatal conductance and transpiration rates of pot-grown Lizixiang and ND98 leaves were measured under normal and saline conditions. The stomatal conductance and transpiration rate were lower in ND98 than in Lizixiang under normal conditions, and these parameters were considerably reduced in ND98, compared to those in Lizixiang, under salt stress (Fig. 7A,B). The contents of JA and ABA were measured in the leaves of the corresponding pot-grown Lizixiang and ND98 plants; the JA content was significantly higher in ND98 than in Lizixiang after salt stress, whereas the ABA content was similar between the two genotypes after salt stress (Fig. 7C,D). Stomatal opening- and closure-related genes in the DEGs were analyzed. Root phototropism protein 2 (*IbRPT2*), which is involved in stomatal opening, exhibited significantly lower expression (Fig. 7E, Supplementary Table S6), whereas transcription factor *IbbHLH93,* which leads to a weak stomatal phenotype, showed significantly higher expression in ND98 than in Lizixiang (Fig. 7F, Supplementary Table S6). These results indicated that the stomatal closure enhancement in response to the increased JA content and the response of stomatal closure-related genes improved water conservation, stabilized osmotic pressure and reduced membrane damage in the leaves of ND98 under salt stress.

### Analysis of Na^+^ content

The ion transport-related genes potassium transporter 1 (*HKT1*), Na^+^/H^+^ antiporter 1(*NHX1*) and Na^+^/H^+^ antiporter 18 (*NHX18*) were up-regulated in ND98 roots, but down-regulated in Lizixiang roots under salt stress (Supplementary Table S6). The maintenance of a high intracellular K^+^/Na^+^ ratio is important for avoiding Na^+^ poisoning^44^. The Na^+^ and K^+^ contents and the K^+^/Na^+^ ratios in the roots of pot-grown Lizixiang and ND98 were measured under normal and salt conditions. Similar Na^+^ and K^+^ contents were observed in Lizixiang and ND98 under conditions where salt stress was not present, while Na^+^ accumulation was significantly reduced and the K^+^ content and K^+^/Na^+^ ratio were significantly increased in ND98 compared with Lizixiang under salt stress (Fig. 7G). These results showed that ND98 exhibited a more balanced ion state than Lizixiang, contributing to the improved adaptability of ND98 to salt stress.

## Discussion

### The JA signalling pathway plays important roles in the response of sweet potato to salt stress

Sweet potato is one of the most widely cultivated root and tuber crops and has considerable potential for cultivation on saline land. However, the current understanding of sweet potato responses to salt stress remains limited. Plant roots alter their developmental process to rapidly adapt to salt stress. The salt tolerance of sweet potato is improved through increasing betaine, proline and ABA contents, activating ROS scavenging and enhancing myo-inositol biosynthesis, photosynthesis and ion homoestasis^29^,^30^,^31^,^32^,^45^,^46^,^47^,^48^,^49^,^50^.

**Figure 8:**
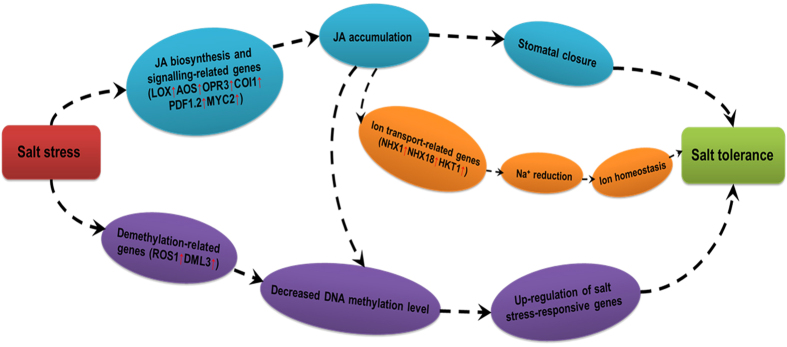
Model of the regulatory networks activated in response to salt stress in ND98.

JA, which is naturally synthesized by plants, plays an important role as a signal molecule that induces tolerance mechanisms under the influence of abiotic stresses^4^,^5^,^6^,^7^,^8^,^9^,^10^,^11^,^51^. However, the roles of the JA signalling pathway in response to abiotic stresses in sweet potato have not been reported to date. In the present study, the salt-sensitive variety Lizixiang and the salt-tolerant line ND98 were used to identify the genes and pathways involved in the salt tolerance of sweet potato via high-throughput RNA-sequencing technology. The DEGs between the plants included a number of genes involved in plant hormone synthesis and signalling (Fig. 2B). The ABA and SA biosynthesis and signalling genes showed changes, but most of these genes were not induced in both genotypes under salt stress. Significant differences were also not observed in the ABA and SA contents in the leaves and roots of Lizixiang and ND98 before and after salt treatments (Fig. 6A,B). Interestingly, the expression levels of the JA biosynthesis and signalling genes (*IbLOX, IbAOS, IbAOC, IbOPR3, IbCOI1* and *IbPDF1.2*) and the content of JA were significantly higher in ND98 than in Lizixiang under salt stress (Fig. 5B). These results indicate that the salt tolerance in ND98 is related to JA content, suggesting that the JA signalling pathway plays important roles in the response of sweet potato to salt stress (Fig. 8).

### JA signalling may regulate stomatal closure in sweet potato

Plants show enhanced stomatal closure and maintain osmotic pressure in response to abiotic stresses^52^. JA stimulates stomatal closure and thus prevents dehydration under salt and drought stresses^53^. Stomatal conductance and transpiration rates are negatively correlated with stomatal closure, and transpiration rates provide an important index to measure the loss of water from leaves^43^. In the present study, the stomatal conductance and transpiration rates were strongly reduced in JA-sufficient ND98 plants, compared with those in Lizixiang plants, under salt stress (Fig. 7A,B,C). Therefore, a lower degree of JA-induced stomatal closure was observed in ND98 than in Lizixiang, and reduced transpiration rates led to decreased leaf water losses in ND98. In addition, the stomatal opening-related gene *IbRPT2* was significantly down-regulated and the stomatal closure-related transcription factor *IbbHLH93* was significantly up-regulated in ND98 (Fig. 7E,F), consistent with the results of Inada *et al*.^54^ and Nadeau *et al*.^55^. These results indicate that JA regulates stomatal closure and reduces leaf water losses, thereby improving the salt tolerance of ND98 (Fig. 8).

### JA and the ion transport network may contribute to the salt tolerance of sweet potato

High soil salinity imposes ion toxicity on plants, in addition to osmotic stress, leading to cell damage and growth arrest. The treatment of salt-stressed plants with exogenous JA has been shown to improve Na^+^ exclusion by decreasing Na^+^ uptake at the root surface^7^,^56^. The ion transport-related gene *HKT1* plays a key role in protecting against Na^+^ toxicity through the promotion of K^+^ accumulation^44^, and another gene, *NHX*1, plays an important function in transporting Na^+^ into the vacuole^57^. The expression of *MaNHX* genes is induced by MeJA^58^, and the signalling network involving MeJA and ion transport controls plant growth under salt stress.

In the present study, the ion transport-related genes *IbHKT1, IbNHX1* and *IbNHX18* were significantly up-regulated and the Na^+^ content was significantly reduced in JA-sufficient ND98 roots, whereas these genes were significantly down-regulated and the Na^+^ content was significantly increased in Lizixiang roots under salt stress (Fig. 7G, Supplementary Table S6). These results suggest that the increased JA content induces the expression of ion transport-related genes and maintains a relative ion balance, thereby improving the salt tolerance of ND98 (Fig. 8).

### Decreased DNA methylation may up-regulate salt stress-responsive genes in sweet potato

Environmental stresses induce changes in global gene expression via DNA methylation or demethylation, with hypermethylation leading to gene down-regulation and hypomethylation leading to gene up-regulation^59^,^60^,^61^. The high expression of the cold stress-related gene *ZmMI1* is associated with a reduction in methylation under cold stress in maize roots^62^, and the hypomethylation of genomic DNA has been reported to lead to the up-regulation of stress-responsive genes in tobacco plants under tobacco mosaic virus (TMV) treatment^63^. Key demethylation-related gene *ROS1* initiates the erasure of 5-methylcytosine through a base excision repair process, and *DML3* is required to remove DNA methylation marks from improperly methylated cytosine and repairs high methylation levels in properly targeted sites^64^. In the present study, the expression levels of demethylation-related genes *IbROS1* and *IbDML3* were significantly higher in ND98 than in Lizixiang (Fig. 4B), and ND98 exhibited reduced methylation compared with Lizixiang under salt stress (Table 2). The proportion of genes with FPKM ≥ 1 was 13.9 and 15.2% higher under the 12- and 48-h salt treatments, respectively, in ND98 than in Lizixiang (Fig. 1B). This result was consistent with the low methylation level of ND98 under salt stress. These results demonstrate that decreased DNA methylation may up-regulate salt stress-responsive genes in sweet potato, which also contributes to the salinity tolerance of ND98 (Fig. 8). The decreased DNA methylation may be caused by the increased JA level and the further study is needed^65^,^66^.

In conclusion, for the first time, the transcriptome of sweet potato under salt stress was analyzed using the salt-sensitive variety Lizixiang and the salt-tolerant line ND98. The results of the transcript profile analysis have revealed that the JA signalling pathway plays important roles in the responses of sweet potato to salt stress, and the increased JA level enhances the salt tolerance of sweet potato by regulating stomatal closure and maintaining ion homeostasis. Decreased DNA methylation may also contribute to the salt tolerance of this crop. These results provide insights into the mechanisms and genes involved in the salt tolerance of sweet potato.

## Methods

### Plant materials

The salt-sensitive sweet potato variety Lizixiang and the salt-tolerant sweet potato line ND98 were used in the present study. For the RNA-Seq analysis, the plants were grown in MS medium at 27 ± 1 °C for 13 h/day under cool-white fluorescent light at 54 μM/m^2^/s for 4 weeks and were subsequently cultivated in Hoagland solution with 200 mM NaCl for 0, 12 and 48 h according to our previous studies^30^,^31^,^32^,^50^. The roots were excised, immediately frozen in liquid nitrogen and subsequently stored at −80 °C until further use. Two independent biological replicates were performed.

### RNA extraction, library construction and RNA-Seq

Total RNA was extracted from the roots of Lizixiang and ND98 plants using the RNAprep Pure Plant Kit (Tiangen Biotech, Beijing, China). The purified RNA concentrations were quantified using a spectrophotometer (UV-Vis Spectrophotometer, Quawell Q5000, San Jose, CA, USA), and the integrity of the RNA samples was examined using an Agilent 2100 Bioanalyzer (Agilent Technologies, Santa Clara, CA, USA).

The RNA-Seq library was constructed using the Illumina TruSeq RNA Sample Preparation Kit (Illumina Inc., San Diego, CA, USA). Poly-A mRNA was isolated from the total RNA samples using poly-T oligo-attached magnetic beads. The mRNA was fragmented using an RNA fragmentation kit (Ambion, Austin, TX, USA) prior to cDNA synthesis to avoid priming bias. The cleaved RNA fragments were transcribed into first-strand cDNA using reverse transcriptase and random primers followed by second-strand cDNA synthesis using DNA polymerase I and RNase H (Invitrogen, Carlsbad, CA, USA). The fragments were ligated to sequencing adaptors and sequenced on an Illumina HiSeq^TM^ 2500 platform.

### Transcript assembly and differential expression

The Trinity method was used for the *de novo* assembly of clean read data^67^. The contigs were linked to transcripts according to the paired-end information of the sequences, and the transcripts were subsequently clustered based on their nucleotide sequence identity. The longest transcripts in the cluster units were regarded as unigenes to eliminate redundant sequences, and the unigenes were subsequently combined to produce the final assembly used for annotation. The transcripts were calculated and normalized to FPKM, representing the gene expression level^68^. DESeq software was used to identify DEGs in pair-wise comparisons. The sequences were deemed significantly differentially expressed at FDR < 0.01, with at least a two-fold change in FPKM.

### Gene annotation

Using BLAST (e < 1e-5) and HMMER (e < 1e-10), the assembled non-redundant transcripts sequences were aligned to various databases, including the NCBI non-redundant (Nr) protein and nucleotide (Nt) databases, the Swiss-Prot protein database, the Cluster of Orthologous Groups (COG) database, the GO database, the euKaryotic Clusters of Orthologous Groups (KOG) database, the KEGG pathway database, the Translated EMBL Nucleotide Sequence Database (TrEMBL) and the protein families (Pfam) database, and Sweetpotato GARDEN^33^. When parsing the alignment results, we assigned the functional description of the top 1 hit with the highest homology to annotate the transcripts in sweet potato.

### Analysis of DNA methylation

The total genomic DNA of the sweet potato roots was extracted using the cetyltrimethylammonium bromide (CTAB) method, and the quality and concentration of DNA were measured using agarose gel electrophoresis (1%) and spectrophotometric assays.

MSAP analysis was performed to detect methylation-sensitive restriction sites in sweet potato root samples using twenty *EcoRI* + *HpaII*/*MspI* primer combinations (Supplementary Table S8) according to the protocol of Gupta *et al*.^69^. The experiments were repeated twice. The polymorphic and well-resolved bands were scored on the basis of the presence (1) or absence (0) of bands of the amplified DNA fragments and were subsequently translated into a data matrix. Four different patterns of PCR amplifications reflected the methylation status and level at the CCGG sites: 1/1, unmethylated (pattern *HpaII*/*MspI* = 1/1); 0/1, fully methylated (pattern *HpaII*/*MspI* = 0/1); 1/0, hemi-methylated (pattern *HpaII*/*MspI* = 1/0); and 0/0, either full methylation or nonexistence of the site (pattern *HpaII*/*MspI* = 0/0). Specific primers are listed in Supplementary Table S8.

### Expression analysis of key genes involved in JA, ABA and SA signalling pathways

Roots of plants treated for 0, 1, 6, 12, 24 or 48 h with 200 mM NaCl in Hoagland solution were used to extract total RNA using the RNAprep Pure Plant Kit (Tiangen Biotech, Beijing, China). RNA samples were reverse-transcribed using the Quantscript Reverse Transcriptase Kit (Tiangen Biotech, Beijing, China). The cDNA solution was used as the template for PCR amplification with specific primers (Supplementary Table S8). The sweet potato β-actin gene was used as an internal control (Supplementary Table S8). The amplifications were performed according to Zhai *et al*.^50^.

### Measurements of the related components

The stomatal conductance and transpiration rate in the leaves and the Na^+^ and K^+^ contents in the roots of pot-grown Lizixiang and ND98 plants treated with 150 mM NaCl were measured according to the methods of Wang *et al*.^32^ and Zhai *et al*.^50^. JA and ABA were quantified using an indirect enzyme-linked immunosorbent assay (ELISA) according to Yang *et al*.^70^. SA was estimated using a Systronics single-beam UV-Visible spectrophotometer at a λ max of 300 nm according to Rao *et al*.^71^.

## Additional Information

**Accession codes:** Transcriptome datasets supporting the conclusions of this article are available in the NCBI SRA repository under the accession number SRP092215.

**How to cite this article**: Zhang, H. *et al*. Transcript profile analysis reveals important roles of jasmonic acid signalling pathway in the response of sweet potato to salt stress. *Sci. Rep.* **7**, 40819; doi: 10.1038/srep40819 (2017).

**Publisher's note:** Springer Nature remains neutral with regard to jurisdictional claims in published maps and institutional affiliations.

## Supplementary Material

Supplementary Dataset 1

Supplementary Dataset 2

Supplementary Dataset 3

Supplementary Dataset 4

Supplementary Information

## Figures and Tables

**Table 1:** Numbers of DEGs at different salt-stressed time points in the two genotypes.

Items		Lizixiang		ND98	
		No.	%	No.	%
12 versus 0 h	up-regulated	2,833	46.74	3,500	44.11
	down-regulated	3,228	53.26	4,435	55.89
	total	6,061		7,935	
48 versus 0 h	up-regulated	3,410	49.13	3,255	41.60
	down-regulated	3,531	50.87	4,569	58.40
	total	6,941		7,824	
48 versus 12 h	up-regulated	697	64.18	404	39.11
	down-regulated	389	35.82	629	60.89
	total	1,086		1,033	
Total		8,744		10,413	

**Table 2:** Comparison of DNA methylation levels between Lizixiang and ND98 based on MASP analysis under normal and salt stress conditions.

Individuals	Total sites	Unmethylated CCGG sites (%)	Methylated CCGG sites (%)		
			Total sites	Full-methylated internal cytosine sites	Hemi-methylated external cytosine sites
L0	731	506 (69.22)	225	120 (53.33)	105 (46.67)
L12	744	449 (60.34)	295	158 (53.56)	137 (46.44)
L48	718	452 (62.95)	266	148 (55.64)	118 (44.36)
L0-12-48-Total	2,193	1,407	786	426	360
N0	767	552 (71.96)	215	128 (59.53)	87 (40.47)
N12	858	503 (66.35)	255	106 (41.57)	149 (58.43)
N48	717	466 (64.99)	251	133 (52.99)	118 (47.01)
N0-12-48-Total	2,342	1,521	721	367	354
