# Supplementary material for: Transcript profile analysis reveals important roles of jasmonic acid signalling pathway in the response of sweet potato to salt stress
Source: Sci Rep. 2017 Jan 13;7:40819. doi: 10.1038/srep40819 (PMC5234020; doi:10.1038/srep40819)
Supplement: Supplementary Information [file srep40819-s5.pdf]

Transcript profile analysis reveals important roles of jasmonic acid signalling pathway in the response of sweet potato to salt stress

Huan Zhang[1], Qian Zhang[1], Hong Zhai[1], Yan Li[1], Xiangfeng Wang[1], Qingchang Liu[1,*] & Shaozhen He[1,*]

[1]Beijing Key Laboratory of Crop Genetic Improvement/Laboratory of Crop Heterosis and Utilization, Ministry of Education, China Agricultural University, Beijing 100193, China

[*]Correspondence and requests for materials should be addressed to S. H. (email: sunnynba@cau.edu.cn) and Q. L. (liuqc@cau.edu.cn)

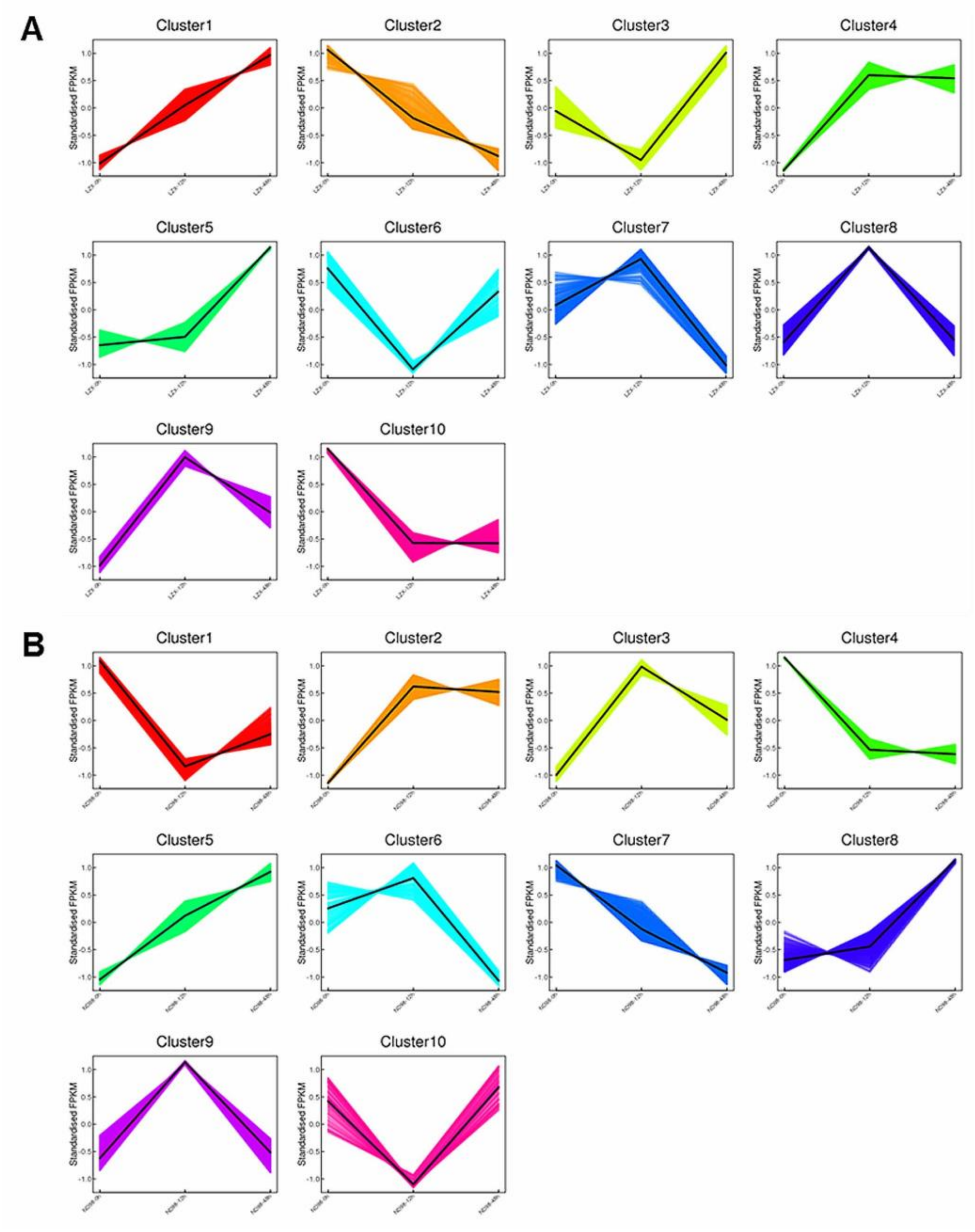

Supplementary Fig. S1. DEG cluster analysis based on the K-means method. The DEGs were divided into 10 categories in Lizixiang (**A**) and ND98 (**B**), respectively, according to their expression patterns.

Supplementary Table S1. Length distribution of the assembled transcripts and unigenes.

Nucleotide length (bp)	Transcript	Unigene
200-300	18,462 (8.01%)	5,871 (9.22%)
300-500	43,511 (18.89%)	22,030 (34.60%)
500-1000	56,596 (24.57%)	17017 (26.73%)
1000-2000	65,110 (25.32%)	11,319 (17.78%)
2000+	46,678 (20.26%)	7,436 (11.68%)
Total number	230,357	63,673
Total length	299,012,560	60,713,683
Mean length	1298.04	953.52

Supplementary Table S2. Statistics of annotated gene information from various databases.

Annotated databases	Unigene	≥ 300 nt	≥ 1000 nt
Annotated in COG	10,351	9,223	6,121
Annotated in GO	18,685	15,883	8,966
Annotated in KEGG	12,240	10,488	6,003
Annotated in KOG	20,059	17,259	10,043
Annotated in Pfam	24,666	22,168	13,946
Annotated in Swiss-Prot	22,764	19,984	11,819
Annotated in TrEMBL	34,086	29,343	16,335
Annotated in Nr	34,689	29,676	16,373
Annotated in Nt	20,746	18,273	12,022
Annotated in at least one database	37,016	31,145	16,623

Supplementary Table S3. The up-regulated, down-regulated and neutral genes in Lizixiang and ND98, respectively. (Supplementary Dataset 1)

Supplementary Table S4. DEGs were higher in ND98 than in Lizixiang for at least one time point during salt treatment. (Supplementary Dataset 2)

Supplementary Table S5. DNA methylation- and demethylation-related genes identified via gene expression profiling. (Supplementary Dataset 3)

Supplementary Table S6. Key genes involved in DNA demethylation and methylation, JA, ABA and SA biosynthesis and signalling, stomatal opening and closure and ion transport.

Gene	ID	FPKM-LZX.0h	FPKM-LZX.12h	FPKM-LZX.48h	FPKM-ND98.0h	FPKM-ND98.12h	FPKM-ND98.48h
**The DNA demethylation-related genes**							
IbROS1	c144343.graph_c0	2.925	1.185	1.395	2.860	6.190	1.820
IbDML3	c148603.graph_c0	26.655	16.360	14.315	58.440	45.285	24.545
**The DNA methylation-related genes**							
IbCMT2	c124784.graph_c0	4.825	4.650	3.805	3.575	5.015	2.050
IbDRM1	c136099.graph_c0	18.515	6.850	11.230	14.860	7.415	10.920
IbDRM2	c156316.graph_c1	49.305	33.700	27.995	42.160	44.825	44.460
IbMET1	c154661.graph_c0	8.433	6.985	6.697	5.720	10.104	11.978
**The JA biosynthesis and signalling-related genes**							
IbLOX	c154376.graph_c0	75.637	184.720	130.424	148.027	298.626	103.040
IbAOS	c110075.graph_c0	2.476	0.389	0.000	5.853	13.274	2.309
IbAOC	c136730.graph_c0	2.989	4.796	7.071	7.091	8.394	9.872
IbOPR3	c151632.graph_c0	10.287	12.939	10.313	4.943	83.780	18.896
IbCOI1	c148337.graph_c2	28.498	8.007	6.102	16.811	14.038	61.087
IbPDF1.2	c136835.graph_c0	128.199	171.131	116.517	493.801	335.206	122.939
IbMYC2	c143913.graph_c2	51.772	22.515	13.868	12.617	11.600	14.959
**The ABA biosynthesis and signalling-related genes**							
IbZEP	c158900.graph_c0	12.184	7.554	4.333	26.565	6.384	3.171
IbAO	c150446.graph_c0	2.290	1.811	1.086	5.547	1.617	0.680
IbNCED	c156411.graph_c0	15.977	25.232	43.713	364.130	47.737	40.918
IbSnRK2	c148195.graph_c0	31.011	165.411	173.425	33.339	111.176	143.886
IbABF1	c125138.graph_c1	21.600	13.545	5.685	13.600	8.770	7.155
**The SA biosynthesis and signalling-related genes**							
IbPAL	c147773.graph_c3	90.323	15.717	13.287	435.560	21.205	16.453
IbNPR1	c151920.graph_c0	8.805	7.100	5.845	14.185	10.470	10.425
IbPR1	c160535.graph_c0	0.313	7.576	5.064	0.163	3.548	7.904
**The stomatal opening and closure-related genes**							
IbRPT2	c158200.graph_c0	161.200	53.980	92.595	33.455	17.985	42.050
IbbHLH93	c152736.graph_c0	27.455	4.165	2.100	3.945	8.060	4.320
**The ion transport-related genes**							
IbHKT1	c158185.graph_c0	24.010	3.490	4.395	19.665	36.235	55.295
IbNHX1	c151428.graph_c0	10.765	10.265	9.580	17.940	14.340	15.400
IbNHX18	c158340.graph_c0	1.030	0.000	0.025	17.075	19.380	29.665

Supplementary Table S7. DEGs from different phytohormone signalling pathways. (Supplementary Dataset 4)

Supplementary Table S8. Primers used in this study.

Primer name	Primer sequence (5′-3′)
Primers for MASP	
EcoRI-adaptor1	CTCGTAGACTGCGTACC
EcoRI-adaptor2	AATTGGTACGCAGTC
HpaII/*MspI*-adaptor1	GATCATGAGTCCTGCT
HpaII/*MspI*-adaptor2	CGAGCAGGACTCATGA
EcoRI-pre-amplification primer	GACTGCGTACCAATTCA
HpaII/*MspI*-pre-amplification primer	ATCATGAGTCCTGCTCGG
EcoRI-AAC	GACTGCGTACCAATTCAAC
EcoRI-AAG	GACTGCGTACCAATTCAAG
EcoRI-ACA	GACTGCGTACCAATTCACA
EcoRI-ACT	GACTGCGTACCAATTCACT
EcoRI-ACC	GACTGCGTACCAATTCACC
EcoRI-ACG	GACTGCGTACCAATTCACG
EcoRI-AGC	GACTGCGTACCAATTCAGC
EcoRI-AGG	GACTGCGTACCAATTCAGG
HpaII/*MspI*-TCT	ATCATGAGTCCTGCTCGGTCT
HpaII/*MspI*-TCG	ATCATGAGTCCTGCTCGGTCG
HpaII/*MspI*-TCC	ATCATGAGTCCTGCTCGGTCC
HpaII/*MspI*-TTC	ATCATGAGTCCTGCTCGGTTC

HpaII/*MspI*-TTG	ATCATGAGTCCTGCTCGGTTG
HpaII/*MspI*-TTA	ATCATGAGTCCTGCTCGGTTA
HpaII/*MspI*-TGA	ATCATGAGTCCTGCTCGGTGA
HpaII/*MspI*-TGT	ATCATGAGTCCTGCTCGGTGT
Primers for qRT-PCR	
β-actin-F	AGCAGCATGAAGATTAAGGTTGTAGCAC
β-actin-R	TGGAAAATTAGAAGCACTTCCTGTGAAC
IbROS1-F	AACACCAGTGGGGAATGTAAGAT
IbROS1-R	ATGATTGCTGTGTTTTGGAGACT
IbDML3-F	CACTCTCCTTCTCCTCCGTCC
IbDML3-R	GAATGGGTTAGTTTCATCGGC
IbLOX-F	GTGCTGAACAACACGCTTTTAG
IbLOX-R	GTTTTAAGGTAGAACTCATTGGGAT
IbAOS-F	GACGGTTTTTGGACATTCAGG
IbAOS-R	GAACGAATCCTGGAGGCACT
IbAOC-F	GACGAGGATTTTGACGAGCA
IbAOC-R	TGGGATAATACAAGCAGCAGGA
IbOPR3-F	CGCCATCCATTCCAAGTTTAG
IbOPR3-R	GGTCCCCCATAATACGCTAAC
IbCOI1-F	CGGCAGTTCTGGAATCTCG
IbCOI1-R	TATGCGAGGAGATGGGCTG
IbPDF1.2-F	GGCTTCATCTCTTCGTTCATTT

IbPDF1.2-R	GCAGTTGCTGTCCCGAGAA
IbMYC2-F	TTTGCCAGAGAAACCTTATCCA
IbMYC2-R	GAGGTTAAACCCATCGCAGG
IbZEP-F	TCGGTTTGAAGTGTTTTCTGTG
IbZEP-R	CAAACTTCTCCTCAAACAACGAC
IbAO-F	GTTACTTGCTATTCTCGGGGTG
IbAO-R	GATGAGTCTTGGCTGGGTTG
IbNCED-F	TTTGGCTAAAGTGGACCTCTCA
IbNCED-R	ATTCCTGGGCAGAAAAAACG
IbSnRK2-F	GGTGCCAGTGATAACCCTCTAA
IbSnRK2-R	CTTGATAGCCACTTTGTGTCCAG
IbABF1-F	GGAGTTTGGGACAGAAAGGAG
IbABF1-R	AAGCCCCGACAGTCACCT
IbPAL-F	TCACTGTGGGTGCTAATGGAG
IbPAL-R	GGCTTGGCGGAGTTTCTG
IbNPR1-F	AGTCCGTTCTTCAGGAGCGT
IbNPR1-R	TTCCGCAATAAAGGTAAGCC
IbPR1-F	GCAAGATTACCTAAACCCCCA
IbPR1-R	GGAGTTGGCGTAGTTCTGCG
IbPRT2-F	GGGAGTAGTGCGAGTGTGAGG
IbRPT2-R	AAAGGATTCAACTTCCCCAACT
IbbHLH93-F	GAGAAATGAGTCGTGGGAAGG

IbbHLH93-R	AAAACAAAAGGCGGAGGC
